# Beneficial effects of a multidomain cognitive rehabilitation program for traumatic brain injury–associated diffuse axonal injury: a case report

**DOI:** 10.1186/s13256-020-02591-7

**Published:** 2021-01-30

**Authors:** Tania de la Rosa-Arredondo, José Alberto Choreño-Parra, Jessica Amira Corona-Ruiz, Patricia Emilia Rodríguez-Muñoz, Francisco Javier Pacheco-Sánchez, Alberto Iván Rodríguez-Nava, Gabriela García-Quintero, Parménides Guadarrama-Ortiz

**Affiliations:** 1 Neuropsychology Department, Centro Especializado en Neurocirugía, Neurociencias México (CENNM), Mexico City, Mexico; 2 Department of Neurosurgery, Centro Especializado en Neurocirugía y Neurociencias México (CENNM), Mexico City, Mexico; 3 Escuela Nacional de Medicina y Homeopatía, Instituto Politécnico Nacional, Mexico City, Mexico

**Keywords:** Cognitive rehabilitation, Traumatic brain injury, Diffuse axonal injury, Cognitive impairment

## Abstract

**Background:**

Neuropsychological rehabilitation is a crucial component of medical care for patients with diffuse axonal injury (DAI). However, current cognitive intervention programs directed to favor the training of specific domains individually have shown controversial results. Here, we evaluated the effectiveness of a neuropsychological rehabilitation program directed to favor training of attention, memory, visuospatial abilities, and executive functioning together in a patient with severe traumatic brain injury (TBI)-associated DAI.

**Case presentation:**

A 26-year-old Hispanic woman with a recent history of a severe TBI attended our center complaining of memory problems, dysarthria, and difficulty in planning. A comprehensive cognitive assessment revealed dysfunction in sustained, selective, and divided attention, alterations in memory, planning, and organization of executive behavior, as well as impairment of visuospatial cognitive functions. The patient underwent a 24-week neuropsychological rehabilitation program directed to favor attention, memory, visuospatial abilities, and executive functioning together. After the cognitive intervention, we observed a better patient's performance in tasks requiring sustained, selective, and divided attention, improvement of encoding and retrieval memory problems, use of spatial relationships, planning, and organization of behavior skills. We also observed generalization effects on other domains, such as learning, mental flexibility, inhibition functions, and language.

**Conclusions:**

In conclusion, our results suggest that neuropsychological rehabilitation programs favoring multiple domains together are useful in reestablishing cognitive deficits in patients with severe DAI.

## Introduction

Cognitive deficits caused by diffuse axonal injury (DAI) contribute to disability observed after traumatic brain injury (TBI). Memory, executive functioning, and speed-of-processing are the main domains affected by DAI, due to alteration of important white matter structures which disrupts brain connectivity [1]. Cognitive rehabilitation aims to favor recovery and compensation of affected functions based on the principles of brain neuroplasticity [2]. Nonetheless, the beneficial effects of cognitive interventions for DAI are controversial [3, 4]. These controversies are, in part, due to an incomplete understanding of the impact of different patients' characteristics on rehabilitation effectiveness. Furthermore, the most beneficial components of cognitive interventions for DAI and the effect of domain-specific vs. multidomain training approaches on general cognitive functioning remain unclear.

## Case presentation

We conducted a single-case study to evaluate the effectiveness of a neuropsychological rehabilitation program directed to favor attention, memory, visuospatial abilities, and executive functioning together, in a patient with severe TBI-associated DAI, using a pretest-posttest design.

**Fig. 1:**
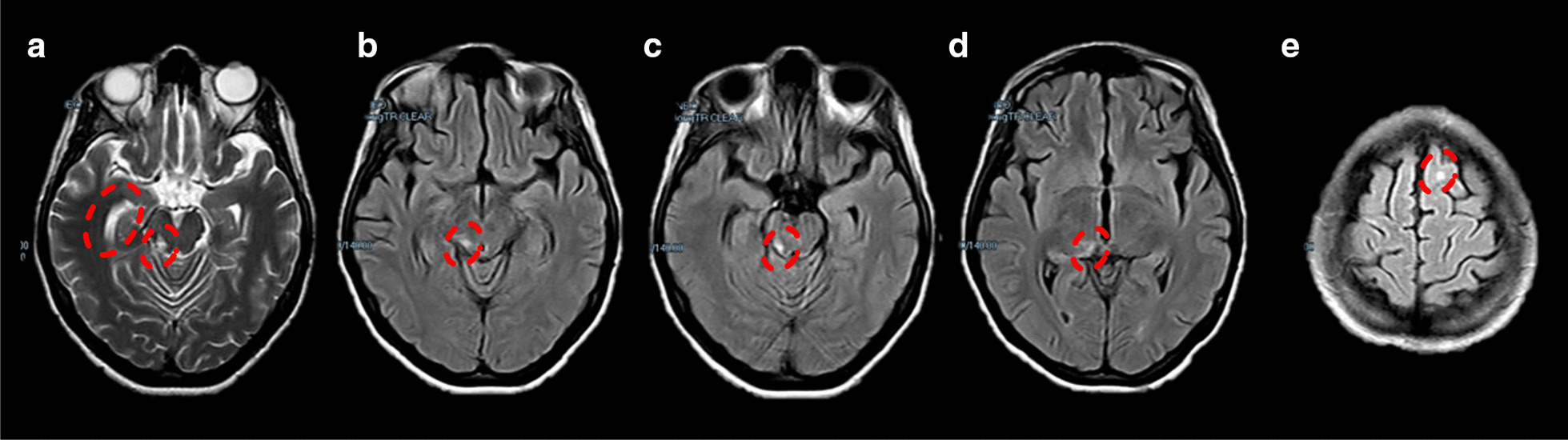
Brain magnetic resonance imaging findings in a patient with diffuse axonal injury. Axial T2-weighted (**a**) and fluid-attenuated inversion recovery magnetic resonance images of the brain (**b**–**e**) showing multifocal hyperintensities (red circles) located at the gray-white matter interface compatible with diffuse axonal injury of grade III

A 26-year-old Hispanic woman attended to our center complaining of memory problems, dysarthria, and difficulty in planning. Nine months earlier, she suffered a severe TBI after a car crash requiring intensive medical care at another hospital. A non-contrasted brain CT scan at admission showed Fisher IV subarachnoid hemorrhage affecting right frontal and parietal sulci, ambiens cisterna, anterior midbrain, pons, medulla oblongata, and a proximal portion of the spinal cord at the level of the foramen magnum. The patient progressively recovered her neurological functioning and was discharged four weeks after injury without a sensitive and motor sequel. A control brain MRI scan revealed multiple non-hemorrhagic focal lesions compatible with DAI of grade III (Fig. 1). However, the patient did not receive any medical follow-up nor neuropsychological rehabilitation during the following months before attending our center. During such a period, she tried to return to her usual professional activities but noticed a diminished performance in tasks that did not represent a challenge before the trauma.

Besides the recent TBI, her past medical history was no relevant. She had a high level of education (master of forensic science). On admission, the patient was evaluated by a clinical neurologist and a neurosurgeon, which did not detect any physical examination abnormality. Then, she was assessed for deficits in several cognitive domains using various neuropsychological tests (Table**1**). During the pre-intervention assessment, the patient was awake, alert, and oriented to person, place, and time. Evaluation of impressive and expressive language revealed dysarthria. Dysfunction in sustained, selective, and divided attention and a decrease in attentional volume and speed-of-processing were found. She presented alterations in memory explained by difficulties in encoding due to attentional deficits aforementioned and showed problems in memory retrieval due to low usage of organizational strategies for learning. We found alterations in the planning and organization of executive behavior, as well as in auditory working memory in its central executive component, which were more evident during tasks demanding a higher level of concentration. Finally, difficulties in managing spatial relationships and coordinates were also observed.

**Table 1:** Neuropsychological test battery employed for cognitive assessment after diffuse axonal injury

Test	Cognitive function assessed	References
NEUROPSI	Orientation, attention, language, memory	[12]
Token test	Language (comprehension)	[13]
Wisconsin Card Sorting Test	Abstract reasoning, concept formation, mental flexibility	[14]
Tower of London (Drexel University version)	Executive functioning (planning, solving problems)	[15, 16]
Barcelona test (cubes, arithmetic, similarities)	Constructive praxis, visuospatial abilities (cubes); numerical processing and calculation (arithmetic); qualities of thinking (similarities)	[17]
Paced Auditory Serial Addition Test (PASAT)	Working memory, speed of processing	[18]

**Fig. 2:**
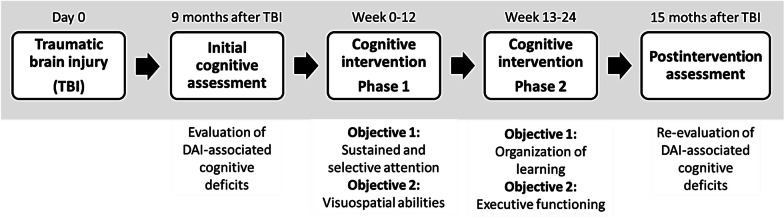
A multidomain cognitive rehabilitation program for a patient with severe traumatic brain injury-associated diffuse axonal injury

Then, the patient underwent a two-phase 24-session cognitive intervention program (Fig. **2**). Phase 1, which had a duration of 12 weeks, was directed to sustained selective auditory and visual attention through tasks of cancelation and counting of different verbal and visual elements. Briefly, the patient had to select among different proposed strategies of verbalization and organization of such elements in order to complete each task correctly. This phase also favored visuospatial and visuoconstructive skills using tasks of copying symmetrical drawings, mosaics, tangram, and copying drawings in grids. Phase 2, which lasted another 12 weeks, favored memory, specifically in the encoding stage, through tasks requiring recall of stories, learning of word lists, taking errands, and reading news. Different strategies were proposed to improve the organization of information to be encoded. These included the division of a story into paragraphs or the classification of written information by semantic groups. We trained selective memory by asking the patient to write a sentence consisting of a pronoun, noun, and adjective summarizing the content of each paragraph of a text. Phase 2 also focused on executive functions such as planning and organizing using labyrinth tasks. Also, we used cards with questions like "what do I have to do?" and "how am I going to do it?" which had to be answered by the patient before executing certain activities.

The patient was able to complete all phases and sessions of the intervention program after 24 weeks of follow-up (15 months after trauma). We found a general improvement of the patient's performance during the postintervention cognitive assessment (Table 2). Specifically, we observed better performance in tasks requiring sustained, selective, and divided attention. Moreover, the attention volume increased, allowing the patient to record all the information presented to her. The encoding and retrieval memory problems significatively improved, and the patient used strategies for organizing information that promoted learning. She also improved her usage of spatial relationships and coordinates, allowing constructive task solving. We found a modest improvement in the planning and organization of behavior skills. Improvement in visual-spatial working memory was observed; however, in terms of auditory working memory, differences in patient performance were discrete.

**Table 2:** Effects of the cognitive intervention program

Cognitive function (test)	Pretest		Posttest		Change after intervention
	Score/result	Performance	Score/result	Performance	
Orientation (Np) Time Space Person	11NS 10 NS 10 NS	Normal Normal Normal	11 NS 10 NS 10 NS	Normal Normal Normal	Constant Constant Constant
Sustained attention (Np)	12 NS	Normal	12 NS	Normal	Constant
Selective attention (Np)	6 NS	Mild to moderate deficit	12 NS	Normal	Improved
Verbal fluency (semantic)	5 NS	Mild to moderate deficit	7 PN	Normal	Improved
Verbal fluency (phonologic)	6 NS	Mild to moderate deficit	8 NS	Normal	Improved
Auditive working memory (PASAT)	Correct responses: 3 Interstimulus interval: <10th percentile	Low	Correct responses: 3 Interstimulus interval: <10th percentile	Low	Constant
Visual working memory (Np, Bt regressing cubes)	3 NS	Severe deficit	12 NS	Normal	Improved
Planning and organizing (TOL DX)	Movements: 1st percentile Start time: 72nd percentile Execution time: 1st percentile Total time: 1st percentile	NA	Movements: 30th percentile Start time: 56th percentile Execution time: 1st percentile Total time: 1st percentile	NA	Improved Improved Constant Constant
Mental flexibility (WCST)	Correct responses: 20th percentile Perseverations: 80th percentile	Low Medium	Correct responses: 50th percentile Perseverations: 70th percentile	Medium Medium	Improved Constant
Inhibition (Np, Stroop interference)	2 NS	Severe deficit	12 NS	Normal	Improved
Visuoconstruction (Bt, cubes)	Correct responses: <10th percentile Time: <10th percentile	Inferior Inferior	Correct responses: 95th percentile Time: 50th percentile	Maximum Medium	Improved Improved
Auditive memory and learning (Np)	Encoding: 10 NS Retrieval: 7 NS	Normal Normal	Encoding: 13 NS Retrieval: 12 NS	Normal Normal	Constant Constant
Visual memory and learning (Np)	Encoding: 7 NS Retrieval: 7 NS	Normal Normal	Encoding: 2 NS Retrieval: 12 NS	Severe Normal	Worsened^a^ Constant^a^
Abstract reasoning (Bt, similarities)	10th percentile	Minimum	10th percentile	Minimum	Constant
Numerical calculation (Bt, arithmetic)	Correct responses: <10th percentile Time: <10th percentile	Low Low	Correct responses: <10th percentile Time: <10th percentile	Low Low	Constant Constant

^*^Despite no quantitative improvement in the normalized score, the patient improved in the performance of the specific tasks as she processed information by clusters at the initial evaluation. However, after the intervention program, she performed the same tasks with better planning abilities allowing her to memorize information. Bt, Barcelona test; NA, not applicable; Np, NEUROPSI; NS, normalized score; PASAT, Paced Auditory Serial Addition Test; TOL DX, Tower of London Drexel University version; WCST, Wisconsin Card Sorting Test

Finally, we scheduled a follow-up appointment at our outpatient clinic to evaluate the general condition of the patient six months after the end of the cognitive intervention. During such an evaluation, the patient scored average in the Mini-Mental State Examination (MMSE) test and the Montreal Cognitive Assessment (MoCA) test. Also, she reported the recovery of her independence and return to her professional activities. The patient provided written informed consent for publication of the case.

## Discussion

Cognitive decline is one of the most critical factors contributing to the disability observed among patients that suffered a TBI after hospital discharge [5]. As such, neuropsychological interventions aimed to recover the affected cognitive functions or promote compensatory mechanisms have increasingly become part of the medical care for patients with TBI. Thus far, neuropsychological rehabilitation programs for patients with TBI-associated DAI have been directed to individual cognitive domains, showing controversial effects and minimal generalization to other domains. For instance, some investigations have proven the effectiveness of attention training in TBI patients, whereas others showed no improvement of attention after rehabilitation [3, 4]. Likewise, poor support exists for memory and speed-of-processing training after TBI [3]. Despite no apparent results, recent data suggest that cognitive rehabilitation significantly modify cerebral activation in patients with TBI [6]. Furthermore, factors like age, cognitive reserve, severity and time postinjury, neurological sequelae, and the approach of intervention influence the neuropsychological rehabilitation effectiveness [4, 7, 8].

In this context, a previous study has shown that training of multiple cognitive domains together, rather than individually, might be beneficial for the recovery of specific and generalized cognitive functions in young patients with mild-to-moderate TBI-associated DAI [9]. Here, we demonstrated that the benefits of this approach might extend to young patients with severe injury. Indeed, although our program focused on attention, memory, visuospatial abilities, and executive functioning, we also observed an improvement in learning, mental flexibility, inhibition functions, and language deficits. The young age and high cognitive reserve of the patient could have contributed to our intervention's effectiveness, as reported before [10]. However, a significant limitation of our study is the absence of a control group. This caveat did not allow us to discriminate between our rehabilitation program's effects and those attributed to a natural recovery process. This limitation is fundamental, as it is expected that patients that suffered a TBI will recover a certain degree of cognitive functioning over time without any intervention [8].

Despite this, we should note that some facts make us believe that the improvements observed here are not likely attributed to a natural recovery process. First, our cognitive intervention started nine months after the TBI. During the intermediate period, the patient did not notice any improvement in her cognitive functioning, which is why she looked for medical care. This contrasts with the dynamics of the natural recovery process of cognitive functions after a TBI, as most patients recover particular abilities early during the first few weeks [11].

Secondly, the cognitive recovery pattern observed in our patient also differed from the natural longitudinal trajectories of neuropsychological functions described after TBI. For instance, visuospatial and executive functioning have shown a linear improvement during the first 15 months after injury in patients with moderate to severe TBI [8]. Conversely, as aforementioned, our patient did not report any improvement of these functions during the first nine months after the trauma. Also, she presented severe impairment in planning, organization of executive behavior, and difficulties in managing spatial relationships and coordinates immediately before receiving cognitive rehabilitation. These cognitive deficits greatly improved after we implemented our multidomain cognitive intervention program.

On the other hand, the literature shows that the overall cognitive functioning recovers most rapidly in patients with mild TBI, returning to baseline within 12 weeks. Cognitive functioning slightly improves after moderate-severe TBI but remains impaired even two years after injury [11]. In contrast, our findings demonstrate that our cognitive rehabilitation program was useful for recovering several cognitive functions even when our patient suffered from a severe injury, and despite our intervention was not delivered immediately after the trauma. Thus, it is highly likely that the patient's cognitive functioning at the end of the rehabilitation (15 months after TBI) was much better than if no intervention would have been provided. However, it is also possible that the delay in establishing our cognitive intervention could ameliorate its effectiveness. In other words, our patient could have obtained a more significant benefit if the rehabilitation would have been provided earlier. Future studies should compare the effectiveness of our rehabilitation program administered early and late after TBI.

Finally, another study limitation is that, due to our single-case design, we cannot affirm that our program is adequate and useful for other TBI groups, such as older patients. Despite these, our findings reinforce the notion that neuropsychological rehabilitation programs directed to multiple, rather than individual cognitive domains, are useful in reestablishing cognitive deficits in young patients with TBI-associated DAI.

## Conclusions

In conclusion, our study describes a novel cognitive rehabilitation program directed to train multiple cognitive domains together in patients with DAI. Although this program is designed to train attention, memory, visuospatial abilities, and executive functioning in a period of 24 weeks, the results obtained here demonstrated generalization effects to other domains, such as learning, mental flexibility, inhibition functions, and language. Thus, our study adds evidence in favor of training multiple cognitive domains together, rather than individually, in young patients with cognitive sequel after TBI-associated DAI.

## Data Availability

The clinical data from the case presented here are available from the corresponding author on reasonable request.

## Abbreviations

Bt: Barcelona test
CT: Computed tomography
DAI: Diffuse axonal injury
MRI: Magnetic resonance imaging
Np: NEUROPSI
PASAT: Paced Auditory Serial Addition Test
TBI: Traumatic brain injury
TOL DX: Tower of London Drexel University version
WCST: Wisconsin Card Sorting Test

## References

[1] Kinnunen KM, Greenwood R, Powell JH, Leech R, Hawkins PC, Bonnelle V, Patel MC, Counsell SJ, Sharp DJ. White matter damage and cognitive impairment after traumatic brain injury. Brain. 2011;134:449–63. 10.1093/brain/awq347 PMC3030764 21193486

[2] Freire FR, Coelho F, Lacerda JR, da Silva MF, Gonçalves VT, Machado S, Velasques B, Ribeiro P, Basile LFH, Oliveira AMP, *et al.* Cognitive rehabilitation following traumatic brain injury. Dement Neuropsychol. 2011;5:17–25. 10.1590/S1980-57642011DN05010004 PMC5619134 29213715

[3] Virk S, Williams T, Brunsdon R, Suh F, Morrow A. Cognitive remediation of attention deficits following acquired brain injury: A systematic review and meta-analysis. NeuroRehabilitation. 2015;36:367–77. 10.3233/NRE-151225 26409340

[4] Rohling ML, Faust ME, Beverly B, Demakis G, *et al.* Effectiveness of cognitive rehabilitation following acquired brain injury: a meta-analytic re-examination of Cicerone *et al.*’s (2000, 2005) systematic reviews 005) systematic reviews. Neuropsychology. 2000;2009(23):20–39. 10.1037/a0013659 19210030

[5] Scheid R, Walther K, Guthke T, Preul C, von Cramon DY. Cognitive sequelae of diffuse axonal injury. Arch Neurol. 2006;63:418–24. 10.1001/archneur.63.3.418 16533969

[6] Galetto V, Sacco K. Neuroplastic changes induced by cognitive rehabilitation in traumatic brain injury: a review. Neurorehabil Neural Repair. 2017;31:800–13. 10.1177/1545968317723748 28786307

[7] Marquez de la Plata CD. Hart T, Hammond FM, Frol AB, Hudak A, Harper CR, O’Neil-Pirozzi TM, Whyte J, Carlile M, Diaz-Arrastia R: impact of age on long-term recovery from traumatic brain injury. Arch Phys Med Rehabil. 2008;89:896–903. 10.1016/j.apmr.2007.12.030 PMC2600417 18452739

[8] Rabinowitz AR, Hart T, Whyte J, Kim J. Neuropsychological recovery trajectories in moderate to severe traumatic brain injury: influence of patient characteristics and diffuse axonal injury. J Int Neuropsychol Soc. 2018;24:237–46. 10.1017/S1355617717000996 PMC5957498 29032776

[9] Alves J, Magalhaes R, Castiajo P, Sampaio A, Goncalves OF, Arantes M. Domain-specific and generalization effects of cognitive intervention in diffuse axonal injury: a case report. J Neuropsychiatry Clin Neurosci. 2012;24:E19-20. 10.1176/appi.neuropsych.11060128 22772682

[10] Green RE, Colella B, Christensen B, Johns K, Frasca D, Bayley M, Monette G. Examining moderators of cognitive recovery trajectories after moderate to severe traumatic brain injury. Arch Phys Med Rehabil. 2008;89:S16-24. 10.1016/j.apmr.2008.09.551 19081437

[11] Schretlen DJ, Shapiro AM. A quantitative review of the effects of traumatic brain injury on cognitive functioning. Int Rev Psychiatry. 2003;15:341–9. 10.1080/09540260310001606728 15276955

[12] Ostrosky-Solis F, Ardila A, Rosselli M. NEUROPSI: a brief neuropsychological test battery in Spanish with norms by age and educational level. J Int Neuropsychol Soc. 1999;5:413–33. 10.1017/s1355617799555045 10439587

[13] De Renzi E, Faglioni P. Normative data and screening power of a shortened version of the Token Test. Cortex. 1978;14:41–9. 10.1016/s0010-9452(78)80006-9 16295108

[14] Barcelo F, Sanz M, Molina V, Rubia FJ. The Wisconsin Card Sorting Test and the assessment of frontal function: a validation study with event-related potentials. Neuropsychologia. 1997;35:399–408. 10.1016/s0028-3932(96)00096-6 9106269

[15] Culbertson WC, Zillmer EA. The Tower of London(DX): a standardized approach to assessing executive functioning in children. Arch Clin Neuropsychol. 1998;13:285–301. 14590643

[16] Shallice T. Specific impairments of planning. Philos Trans R Soc Lond B Biol Sci. 1982;298:199–209. 10.1098/rstb.1982.0082 6125971

[17] Pena-Casanova J, Guardia J, Bertran-Serra I, Manero RM, Jarne A. Shortened version of the Barcelona test (I): subtests and normal profiles. Neurologia. 1997;12:99–111. 9198458

[18] Tombaugh TN. A comprehensive review of the Paced Auditory Serial Addition Test (PASAT). Arch Clin Neuropsychol. 2006;21:53–76. 10.1016/j.acn.2005.07.006 16290063

